# Building a Statistical Model for Predicting Cancer Genes

**DOI:** 10.1371/journal.pone.0049175

**Published:** 2012-11-15

**Authors:** Ivan P. Gorlov, Christopher J. Logothetis, Shenying Fang, Olga Y. Gorlova, Christopher Amos

**Affiliations:** 1 Department of Genitourinary Medical Oncology, The University of Texas MD Anderson Cancer Center, Houston, Texas, United States of America; 2 Department of Cancer Genetics, The University of Texas MD Anderson Cancer Center, Houston, Texas, United States of America; 3 Department of Epidemiology, The University of Texas MD Anderson Cancer Center, Houston, Texas, United States of America; 4 Department of Community and Family Medicine, Geisel School of Medicine, Dartmouth College, Lebanon, New Hampshire, United States of America; National Cancer Institute, National Institutes of Health, United States of America

## Abstract

More than 400 cancer genes have been identified in the human genome. The list is not yet complete. Statistical models predicting cancer genes may help with identification of novel cancer gene candidates. We used known prostate cancer (PCa) genes (identified through KnowledgeNet) as a training set to build a binary logistic regression model identifying PCa genes. Internal and external validation of the model was conducted using a validation set (also from KnowledgeNet), permutations, and external data on genes with recurrent prostate tumor mutations. We evaluated a set of 33 gene characteristics as predictors. Sixteen of the original 33 predictors were significant in the model. We found that a typical PCa gene is a prostate-specific transcription factor, kinase, or phosphatase with high interindividual variance of the expression level in adjacent normal prostate tissue and differential expression between normal prostate tissue and primary tumor. PCa genes are likely to have an antiapoptotic effect and to play a role in cell proliferation, angiogenesis, and cell adhesion. Their proteins are likely to be ubiquitinated or sumoylated but not acetylated. A number of novel PCa candidates have been proposed. Functional annotations of novel candidates identified antiapoptosis, regulation of cell proliferation, positive regulation of kinase activity, positive regulation of transferase activity, angiogenesis, positive regulation of cell division, and cell adhesion as top functions. We provide the list of the top 200 predicted PCa genes, which can be used as candidates for experimental validation. The model may be modified to predict genes for other cancer sites.

## Introduction

A census of human cancer genes conducted by Futreal *et al.* [1] and updated by Santarious *et al*. [2] to identify 400 cancer-related genes. It is obvious that this list of cancer-related genes is not complete: a PubMed search of the literature conducted in June 2011 using the term “novel cancer gene” in the title identified more than 100 papers published in 2011 (data not shown).

Development of a predictive model for cancer genes could accelerate their identification. In this study, we developed a statistical model for the prediction of prostate cancer (PCa) genes. Our study was motivated by the following: i) a number of PCa-related genes with strong experimental evidence have been identified, ii) many genes in the human genome are extensively annotated, and iii) genome-wide profiling of gene-expression data is available [3], [4]. In this study, we identified traits that are characteristic of known PCa genes and used them to predict novel PCa genes.

## Materials and Methods

### Known PCa Genes

We used the KnowledgeNet (KN; a literature-mining algorithm) approach to identify PCa genes [5]. The KN algorithm searches for an association between the gene and both primary and secondary (*i.e.,* related) terms. As a result, each gene receives a confidence score (CS): the higher the CS, the stronger the association of the gene with a specified phenotype; in our case, PCa. We identified a total of 707 genes with CSs ranging from 2.663 to 0.001 (Table S1) and used the top 100 genes as “known PCa genes.” The other 607 genes from the list were considered “putative PCa genes.” The remaining 14,641 genes with a CS<0.001 were considered “non-PCa genes.” We excluded the 607 putative PCa genes to create a well-defined binary outcome for our analysis.

Because our initial search term to identify PCa genes was “prostate cancer,” the algorithm searches the genes associated with any aspects of prostate carcinogenesis, including initiation, progression, recurrence, and survival. In other words, we used a broad definition of PCa genes. Of course, a search can be more specific, *e.g*., “prostate cancer recurrence,” and this is expected to produce a training set that will be different from the list we used.

### Model and Variables

A binary logistic regression (BLR) model was used to discriminate between the “known PCa” and the “non-PCa” genes. Each gene was described by 33 variables (Table 1). The variables were selected on the basis of evidence published by us and others that the individual variables are associated with PCa [6]–[11]. A detailed description of the variables can be found in the Information S1. We subclassified the variables into two categories: prostate-tissue specific and nonspecific. Tissue-specific variables included gene expression data in normal and tumorous prostate tissues. Non–tissue-specific variables were those that can be applied to any type of tissue, *e.g.,* “growth factor,” “phosphorylated” variables.

**Table 1:** Variables used to build a binary logistic model to discriminate known PCa genes.

Type of variable	Variable	Source of the data
Specific	Three-level meta-analysis	Ref. [10]
Nonspecific	Acetylated	GO*
Nonspecific	Angiogenesis	GO
Nonspecific	Antiapoptotic	GO
Nonspecific	Cell adhesion	GO
Nonspecific	Cell proliferation	GO
Nonspecific	Chromatin remodeling	GO
Specific	Difference in expression –LOG(P)	Refs. [12], [13]
Nonspecific	DNA repair	GO
Nonspecific	DNA replication	GO
Nonspecific	Evolutionary conservation index	HomoloGene†
Nonspecific	Expression level in normal prostate	Ref. [25]
Nonspecific	Extracellular space	GO
Nonspecific	Growth factors	GO
Nonspecific	Housekeeping gene	Ref. [26]
Nonspecific	Kinases	GO
Specific	Mean expression in adjacent tissue	Refs. [12], [13]
Specific	Mean expression in tumor tissue	Refs. [12], [13]
Specific	Meta-analysis of the gene expression	Ref. [8]
Nonspecific	Methylated	GO
Nonspecific	Phosphatases	GO
Nonspecific	Phosphorylated	GO
Nonspecific	Plasma membrane	GO
Specific	Prostate-specific expression (enrichment score)	Ref. [25]
Nonspecific	Secreted	GO
Nonspecific	Signal transduction	GO
Nonspecific	Sumoylated	GO
Nonspecific	Transcription	GO
Nonspecific	Transcription factors	GO
Nonspecific	Translation	GO
Nonspecific	Ubiquitinated	GO
Specific	Variance in adjacent tissue	Refs. [12], [13]
Specific	Variance in tumor tissue	Refs. [12], [13]

* GO, Gene Ontology database [27], [28].

† HomoloGene Database: http://www.ncbi.nlm.nih.gov/homologene.

Because our regression model was naturally unbalanced, with too many “non-PCa” genes and too few PCa genes, we could not use a 0.5 threshold to decide whether the gene was a PCa or “non-PCa” gene. The classification threshold (0.05) was chosen to ensure that at least 95% of non-PCa genes were predicted correctly, and because it reflects the proportion of genes that were identified as prostate cancer (707) related to the total number of genes studied in the training phase (14,641). This relatively high rate of correct classification of “non-PCa” genes was selected to reduce the risk of experimental follow-up of false positives, which can be costly.

In total, we used 15,348 genes. Gene expression data were a limiting factor of inclusion of each gene in the analysis. We used the publicly available datasets GSE6919 [12], [13] and GSE21034 [13] from the Gene Expression Omnibus (GEO) [3], [4] and used AmiGO^2^ [14] to identify the genes associated with specific biologic function, cellular location, and posttranslational modifications. The number of human orthologs reported in the HomoloGene database (http://www.ncbi.nlm.nih.gov/HomoloGene) was used as the evolutionary conservation index [15], [16].

### Validation of the Model

To validate the model, we first randomly subclassified the 200 genes with the highest CS into discovery and validation sets. Next we built the BLR model by using only the discovery set and used it to predict PCa genes in the validation set. For additional internal validation, we built the BLR model by using the top 100 genes, excluding the putative PCa genes, and then applied the model to compute the probability for the putative PCa genes. We expected that the probability of being classified as a PCa gene would be higher for the putative genes than it would be for the non-PCa genes. Further, we performed permutation testing by randomly assigning PCa gene status. We built a BLR model for those “mock” PCa genes by using the same set of variables we used for the “real” PCa genes (*i.e*., those identified with KN). We performed this procedure 100 times and estimated the percentage of the correctly predicted PCa genes.

For external validation, we checked to see whether the model-derived probability of a gene’s being PCa related was higher for genes for which recurrent somatic mutations in prostate tumor samples are reported in the Catalogue of Somatic Mutations in Cancer (COSMIC) database [17], [18]. We also used the genes identified as having recurrent somatic mutations in the recently published study results of whole-exome sequencing of prostate tumor samples [19]. Note, however, that we did not use somatic mutation data to build our model.

### Is the Predicting Model Prostate Specific?

To answer this question, we identified the top 100 breast and top 100 lung cancer genes (Table S2) by using the same KN algorithm we used to identify the PCa genes. Then we compared the percentages of correctly predicted breast and lung cancer genes with the percentage of correctly predicted PCa genes.

We built BLR models on the basis of only specific (“specific model”) and nonspecific (“nonspecific model”) predictors. Then we estimated the percentages of correctly predicted non-PCa and PCa genes for each model. Statistical analysis was conducted using SPSS version 15.0.

## Results

### Predicted PCa Genes

Among the 33 variables, 22 were significant in the univariable analysis (Table S3), whereas in the multivariable stepwise-forward (likelihood ratio) BLR model, 16 variables were significant (Table 2). The model correctly predicted 96% of the non-PCa genes and 55% of the PCa genes and was more accurate than the model built on the data that included the putative PCa genes as non-PCa genes, in which 96% of non-PCa genes and 46% of PCa genes were predicted correctly.

**Table 2:** Variables significant in the multivariable binary logistic regression model with putative PCa genes excluded.

Variable	B*	SE	χ^2^	df	*p* Value
Prostate-specific expression(enrichment score)	0.313	0.039	66.116	1	<0.001
Kinases	1.929	0.333	33.647	1	<0.001
Variance in adjacent tissue	0.68	0.131	27.097	1	<0.001
Phosphatases	2.486	0.483	26.469	1	<0.001
Growth factors	1.818	0.453	16.132	1	<0.001
Meta-analysis of thegene expression	0.143	0.037	15.226	1	<0.001
Transcription factors	1.201	0.326	13.562	1	<0.001
Antiapoptotic	1.497	0.415	13.043	1	<0.001
Extracellular space	0.91	0.303	9.05	1	0.003
Signal transduction	0.781	0.272	8.269	1	0.004
Cell proliferation	1.131	0.396	8.154	1	0.004
Ubiquitinated	0.574	0.244	5.542	1	0.019
Angiogenesis	1.062	0.461	5.32	1	0.021
Acetylated	−0.577	0.251	5.276	1	0.022
Cell adhesion	0.804	0.386	4.342	1	0.037
Sumoylated	0.937	0.466	4.043	1	0.044

* B, regression coefficient; SE, standard error.

Table S4 lists the top 200 predicted PCa genes and indicates whether they were known, putative, or novel predicted genes. Ranking the genes according to the model-derived probabilities reshaped the original CS-based list: *AR* (androgen receptor) was ranked seventh, not first, as on the original list, and *KLK3* (prostate-specific antigen [PSA]) was fourth, although it was second on the original list. Overall, the correlation between the CS and the model-derived probability of being PCa related was 0.32, df = 200; *p* = 2×10^–6^. Table S5 shows individual variables contributing to the probability that the gene is associated with PCa.

### Putative PCa Genes have a Higher Probability of being Classified as PCa Related

Putative PCa genes are expected to have a higher probability of being PCa related than non-PCa genes have. We used our model based on the data without the putative genes to estimate the probability that a putative gene is PCa related, comparing the proportions of the genes predicted to be PCa related between the known, putative, and non-PCa genes. The proportions of the genes predicted to be PCa related were 0.052±0.002 for the non-PCa genes, 0.224±0.017 for the putative PCa genes, and 0.547±0.049 for the known PCa genes. As noted earlier, we also built a model that included the putative PCa genes as non-PCa genes. Overall, the prediction accuracy was lower with this model, with the proportions of the genes predicted to be PCa associated being 0.037±0.002 for the non-PCa genes, 0.217±0.016 for the putative PCa genes, and 0.455±0.049 for the known PCa genes.

### Is the Prediction PCa Specific?

To find out whether our predictive model is PCa specific, we identified the top 100 breast and lung cancer genes using the KN-based approach (Table S2). Overall, the proportion of the correctly predicted cancer genes was higher for prostate (0.55±0.03) than for breast (0.37±0.02) and lung cancers (0.31±0.02). For the model built based on nonspecific predictors only, accuracy was better for the PCa genes (0.55±0.02) than it was for the breast (0.24±0.02) and lung cancer (0.21±0.02) genes. And for the model based on specific predictors, the predicting efficiency also was higher for prostate (0.30±0.02) than it was for breast (0.08±0.01) and lung cancer (0.08±0.01) genes.

### Discovery and Validation Sets

For internal validation, we randomly assigned the top 200 PCa-related genes to discovery and validation sets so there were 100 genes in each group. We then built the BLR model on the basis of the discovery set and used it to predict PCa genes from the validation set. The discovery model correctly predicted 95% of the non-PCa genes and 43±5% of the PCa genes; it predicted similar proportions in the validation set: 96% of the non-PCa genes and 38±5% of the PCa genes. We performed this procedure 100 times.

### Permutations

We randomly assigned PCa status to 100 genes from the 15,348 genes in the original table and built a prediction model for those “mock” genes using the same 33 variables (Table 1). The procedure was performed 100 times. There were an average of 0–2 significant variables in the mock gene model, and those variables varied from model to model. On average, 0.7±0.2% mock PCa genes were predicted correctly, which is significantly (*p*<<10^–6^) lower than the percentage of the correctly predicted “true” PCa genes (55±5%).

### External Validation

**Figure 1:**
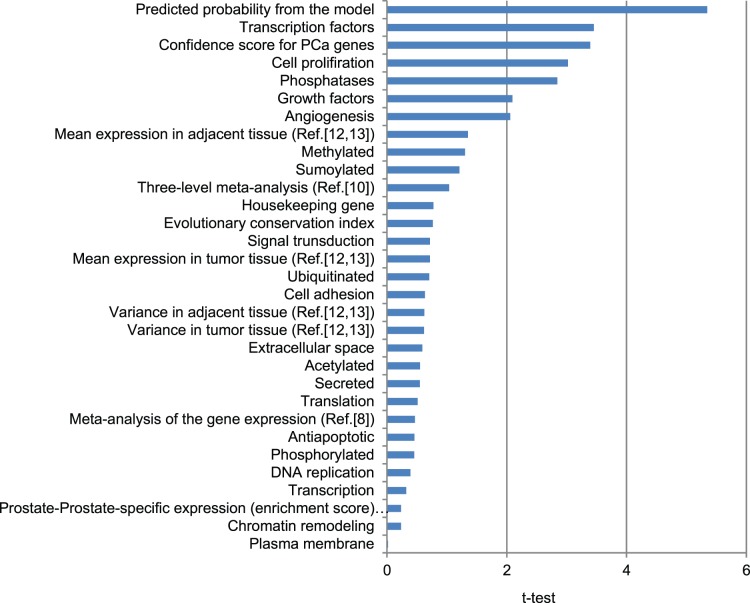
Variables that discriminate genes with recurrent somatic mutations in prostate tumors from all other genes. Vertical line represents a threshold for statistical significance.

For external validation, we used the results of the recently published report on recurrent somatic mutations in prostate tumors [19]. That study identified 20 genes–*BDH1, DKK1, DLK2, FSIP2, GLI1, IKZF4, KDM4B, MGAT4B, NMI, NRCAM, PCDH11X, PDZRN3, PLA2G16, RAB32, SDF4, SF3A1, TBX20, TFG, TP53,* and *ZNF473–*that have recurrent somatic mutations. Seventeen of those genes (all except *BDH1, FSIP2*, and *PLAG16*) were on our original list of 15,348 genes. We found that the model-generated probability of being a PCa gene was more than ten times greater for the genes with recurrent somatic mutations than it was for all the other genes: 0.082±0.041 *vs.* 0.007±0.001; df = 15,348, *t* = 5.4, *p*<10^–6^ (Figure 1). The other significant predictors were transcription factors, the CS used to rank the PCa genes from literature mining, cell proliferation, phosphatases, growth factors, and angiogenesis. We obtained similar results for the genes with the reported PCa somatic mutations from the COSMIC database [18]. The model-derived probability of being a PCa gene was the most significant predictor of genes with recurrent somatic mutations in prostate tumors. Other significant predictors included CS, kinases, antiapoptotic, cell proliferation, acetylated, plasma membrane, and angiogenesis.

### Specific vs. Nonspecific Predictors

We constructed a model based on only specific (eight variables) and only nonspecific (25 variables) predictors. In the nonspecific model, 11 variables were significant (in decreasing order of statistical significance): kinases, phosphatases, extracellular space, transcription factors, antiapoptotic, signal transduction, growth factors, cell proliferation, sumoylated, cell adhesion, and angiogenesis. The nonspecific model correctly predicted 95% of non-PCa and 40% of PCa genes; that based on specific variables correctly predicted 95.5% of non-PCa and 30.2% of PCa genes. There were four significant predictors in that model (in decreasing order of statistical significance): prostate-specific expression (enrichment score), variance in adjacent tissue, meta-analysis of the gene expression, and three-level meta-analysis.

## Discussion

We have identified a combination of traits that is characteristic of PCa genes: a typical PCa gene is a prostate-specific transcription factor, kinase, or phosphatase with high interindividual variance in adjacent normal prostate tissue and is expressed differently (upregulated or downregulated) in normal prostate tissue and primary tumor. PCa genes are likely to have an antiapoptotic effect and play a role in cell proliferation, angiogenesis, and cell adhesion. Their products are likely to be ubiquitinated or sumoylated but not acetylated. They are likely to be involved in signal transduction and being a component of extracellular space. Some of the identified characteristics of PCa genes (*e.g.,* cell proliferation or angiogenesis) are obvious, whereas others (*e.g*., tissue specificity, higher variance of the gene expression in adjacent normal prostate tissue, or ubiquitination) are not that apparent. Because several different factors are involved in nominating a gene to be prostate cancer related, different genes show effects from different predictors. The predictors are indicated in Table S5.

Our model also allows ranking of the genes that are, according to the model-generated evidence, PCa related and therefore predictive of novel PCa genes. A brief description of the top ten novel predicted PCa genes follows.

*UPK3A–*uroplakin 3A; a member of the uroplakin family, a group of transmembrane proteins that form complexes on the apical surface of the bladder epithelium. Mutations in *UPK3A* are associated with renal adysplasia [20].

*KITLG–*encodes the ligand of the tyrosine-kinase receptor. The gene is believed to play a role in cell migration [21].

*NPY–*widely expressed in the central nervous system and influences many physiologic processes, including cortical excitability, stress response, food intake, circadian rhythms, and cardiovascular function.

*GHR–*a member of the type I cytokine receptor family.

*SCGB1A–*a member of the secretoglobin family of small secreted proteins. The encoded protein has been implicated in numerous functions, including anti-inflammation, inhibition of phospholipase A2, and sequestration of hydrophobic ligands.

*NR3C1–*encodes the glucocorticoid receptor, which can function as both a transcription factor and a regulator of other transcription factors.

*JUP–*encodes a protein that is a structural element of submembranous plaques of desmosomes. It forms complexes with cadherins.

*NPM1–*encodes a phosphoprotein that moves between the nucleus and the cytoplasm. The gene product is thought to be involved in several processes, including regulation of the ARF/p53 pathway.

*CD177–*NB1, a glycosyl-phosphatidylinositol–linked *N*-glycosylated cell-surface glycoprotein, was first described in a case of neonatal alloimmune neutropenia [22].

*FAM55D–*chromosome 11 open reading frame 33. Little is known about this gene, but it is downregulated in prostate tumor.

We conducted functional annotation of novel PCa genes by using all 15,348 genes as a background to account for possible selection bias. For the functional annotation, we used the Database for Annotation, Visualization, and Integrated Discovery (DAVID) [23]. The top biologic functions associated with the novel PCa genes were antiapoptosis, regulation of cell proliferation, positive regulation of kinase activity, positive regulation of transferase activity, angiogenesis, positive regulation of cell division, cell adhesion, MAPKKK cascade, bone development, and regulation of cellular localization. (More detailed information can be found in the Supporting Information.) There is considerable overlap between the description of the known and novel predicted PCa genes’ functions: antiapoptosis, regulation of cell proliferation, positive regulation of kinase activity, positive regulation of transferase activity, and MAPKKK cascade are present on both lists. The only unique function associated with the predicted novel PCa genes was bone development in ten genes: *GHR, AMELX, TRAF6, FGF9, SMAD1, CTGF, IGF2, AMBN, FGF18,* and *PTN*.

The results of the internal validation demonstrated that PCa-related genes are not a random collection of genes but rather share a combination of several traits. They also demonstrate that we are unlikely to overfit the model. External validation demonstrated that the model-generated probability of being a PCa gene is the most significant predictor of the PCa candidates identified through the analysis of recurrent somatic mutations. On the other hand, the presence of somatic mutations in tumor samples may be one of the factors that elevate the CS and consequently contribute to the higher chance of being classified as a known PCa gene. Indeed, the CS was the third most significant predictor of the genes with recurrent somatic mutations. However, it was lower than the *t* statistic for the model-generated probability of being a PCa gene: 5.5 *vs.* 3.4. The proportion of the genes with COSMIC somatic mutations was higher among the putative PCa genes: χ^2^ = 22.8, df = 1, *p*<0.0001. The proportion was borderline higher for the predicted novel PCa genes: χ^2^ = 3.8, df = 1, *p* = 0.05. We also found that the average model-derived probability of the published 112 genes with a signature of positive selection [24] was higher than that of an average gene in the human genome: Student’s *t* test = 2.0, df = 30,495, *p* = 0.04. The overlap is modest but significant, especially if we take into account that the published list of the cancer genes was generated for any type of cancer, while in our study we focused on PCa only.

We demonstrated that both specific and nonspecific predictors are important: models based on only specific or only nonspecific predictors are less efficient than the model built on combination of the traits. The specific predictor–based model is more prostate specific than is the model based on nonspecific predictors.

Obviously the structure of the predicting model depends heavily on the training set. We used a broad definition of PCa with the following secondary terms: prostate cancer cells, prostate cancer risk, Gleason, androgen-independent, prostatic neoplasms, Gleason score, prostatectomy, metastatic prostate, human prostate cancer, radical prostatectomy, androgen-independent prostate, advanced prostate, prostate-specific antigen, primary prostate, benign prostate, prostate tumors, prostate-specific, prostate carcinogenesis, and benign prostatic. Although in its current form the model is designed to predict broadly defined PCa genes, it can be adjusted to be more specific; for example, to predict PCa-progression genes. The crucial element here is to define a reliable training set for PCa genes associated with cancer progression.

The BLR model is one of many available classification algorithms. To see whether other classification methods could produce similar results, we also analyzed our data by using linear discriminant analysis (LDA) and support vector machines (SVM). We found that LDA and BLR have rather similar classification efficacies: 51% and 55% correctly classified PCa genes with 95% and 96% of the correctly classified non-PCa genes, with essentially the same set of significant predictors in the model. Validation was also slightly better for the BLR model, with 18% of putative PCa genes predicted to be PCa genes, compared with 22% for LDA model. Compared with the BLR, the SVM was more efficient in the discovery set, correctly predicting 84% of the known PCa genes and 95% of the non-PCa genes; however, in the validation, it correctly predicted only 34% of PCa genes, whereas the BLR model correctly predicted 46% of PCa genes in discovery and 44% in validation set. Because of that better validation efficiency, we focused on BLR model.

The next logical step would be experimental validation of the novel PCa candidates identified by the model. We think that one of the best ways to do that would be with a high-throughput screening platform. For example, one can use high-throughput RNAi screening of PCa cell lines. After silencing of a candidate gene by RNAi, one can estimate the effect of the gene on cell proliferation, migration, and apoptosis. Genes with a strong effect on these cancer-associated phenotypes can be further analyzed in human tissue to confirm their role in prostate tumorigenesis.

In conclusion, we have developed a bioinformatics-based BLR model for prediction of the genes associated with PCa. The model allows ranking human genes according to their probability of being PCa associated. We identified a number of novel PCa candidates with high probabilities of being PCa related, and those candidates may merit further experimental validation. The approach we used can also be applied to other types of genes and other types of cancer; we are currently working on the model for prediction of lung cancer genes.

## Supporting Information

Table S1 The 707 genes with CS ranging from 2.663 to 0.001; we used the top 100 of these genes as “known PCa genes.”
(XLSX)

Table S2 The top 100 breast cancer and 100 lung cancer genes identified by using the KnowledgeNet approach.
(DOCX)

Table S3 Univariable analysis identified 22 of the original 33 original variables as significant predictors of PCa genes.
(DOCX)

Table S4 Ranking of the top 200 genes by model-generated probability of being PCa related. P, putative PCa gene; K, known PCa gene; NP, novel predicted PCa gene.
(DOCX)

Table S5 Individual contributing variables in novel predicted PCa genes. Highlighted variables contribute to a high probability for a gene to be PCa associated. For binary variables, positive contributors have the value of 1; for continuous variables, predictors have a value higher than m+σ, where m is a mean and σ is a standard deviation.
(XLS)

Information S1 Description of the Variables Used to Build the Prediction Model. Variables Are Listed in the Order in Which They Are Presented in Table 1.
(DOCX)
