# Supplementary material for: Building a Statistical Model for Predicting Cancer Genes
Source: PLoS One. 2012 Nov 15;7(11):e49175. doi: 10.1371/journal.pone.0049175 (PMC3499550; doi:10.1371/journal.pone.0049175)
Supplement: Table S2 — The top 100 breast cancer and 100 lung cancer genes identified by using the KnowledgeNet approach. (DOCX) [file pone.0049175.s002.docx]

**Table S2.** The top 100 breast cancer genes identified by using the KnowledgeNet approach.

| Gene symbol | Entrez ID | Confidence score |
| --- | --- | --- |
| *BRCA1* | 672 | 2.617 |
| *BRCA2* | 675 | 1.596 |
| *ERBB2* | 2064 | 1.109 |
| *ESR1* | 2099 | 0.85 |
| *TP53* | 7157 | 0.561 |
| *CYP19A1* | 1588 | 0.427 |
| *CHEK2* | 11200 | 0.39 |
| *ESR2* | 2100 | 0.377 |
| *VEGFA* | 7422 | 0.373 |
| *EGFR* | 1956 | 0.293 |
| *PGR* | 5241 | 0.266 |
| *CCND1* | 595 | 0.261 |
| *AR* | 367 | 0.245 |
| *GSTM1* | 2944 | 0.245 |
| *PTGS2* | 5743 | 0.242 |
| *NAT2* | 10 | 0.226 |
| *PLAU* | 5328 | 0.211 |
| *PPARG* | 5468 | 0.209 |
| *BCCIP* | 56647 | 0.207 |
| *DPYD* | 1806 | 0.196 |
| *NCOA3* | 8202 | 0.19 |
| *GSTT1* | 2952 | 0.18 |
| *MMP2* | 4313 | 0.171 |
| *MUC1* | 4582 | 0.169 |
| *IGFBP3* | 3486 | 0.169 |
| *NRG1* | 3084 | 0.166 |
| *MAPK1* | 5594 | 0.163 |
| *ATM* | 472 | 0.157 |
| *MMP9* | 4318 | 0.153 |
| *CYR61* | 3491 | 0.139 |
| *CDKN1A* | 1026 | 0.138 |
| *KLK10* | 5655 | 0.136 |
| *SYK* | 6850 | 0.134 |
| *IGF1R* | 3480 | 0.131 |
| *CDKN1B* | 1027 | 0.126 |
| *BCL2* | 596 | 0.125 |
| *IGF1* | 3479 | 0.122 |
| *FHIT* | 2272 | 0.121 |
| *PTPRG* | 5793 | 0.121 |
| *SPP1* | 6696 | 0.121 |
| *MEST* | 4232 | 0.12 |
| *CDH1* | 999 | 0.113 |
| *COMT* | 1312 | 0.11 |
| *PRL* | 5617 | 0.109 |
| *FBLN1* | 2192 | 0.109 |
| *RASSF1* | 11186 | 0.108 |
| *PPARA* | 5465 | 0.108 |
| *CYP2E1* | 1571 | 0.107 |
| *TERT* | 7015 | 0.107 |
| *MDM2* | 4193 | 0.105 |
| *ETS1* | 2113 | 0.104 |
| *CYP3A4* | 1576 | 0.103 |
| *SHBG* | 6462 | 0.102 |
| *CD44* | 960 | 0.102 |
| *MYC* | 4609 | 0.1 |
| *TGFB1* | 7040 | 0.1 |
| *TSG101* | 7251 | 0.1 |
| *CYP1A1* | 1543 | 0.1 |
| *BRCA3* | 60500 | 0.099 |
| *TFF1* | 7031 | 0.093 |
| *GJA1* | 2697 | 0.093 |
| *IRS1* | 3667 | 0.092 |
| *GPER* | 2852 | 0.092 |
| *BARD1* | 580 | 0.091 |
| *MTHFR* | 4524 | 0.091 |
| *SERPINB5* | 5268 | 0.091 |
| *BCL6* | 604 | 0.09 |
| *IRS2* | 8660 | 0.088 |
| *CDC2* | 983 | 0.088 |
| *IL18* | 3606 | 0.086 |
| *FAS* | 355 | 0.086 |
| *ADH1B* | 125 | 0.086 |
| *MTA1* | 9112 | 0.085 |
| *IGF2R* | 3482 | 0.084 |
| *CYP1B1* | 1545 | 0.084 |
| *WT1* | 7490 | 0.083 |
| *ITGB3* | 3690 | 0.081 |
| *ABCG2* | 9429 | 0.08 |
| *GSTP1* | 2950 | 0.08 |
| *PCGF2* | 7703 | 0.078 |
| *EBAG9* | 9166 | 0.077 |
| *SNCG* | 6623 | 0.076 |
| *JUN* | 3725 | 0.076 |
| *DUSP3* | 1845 | 0.076 |
| *IL1A* | 3552 | 0.075 |
| *RAC1* | 5879 | 0.075 |
| *HIF1A* | 3091 | 0.074 |
| *NBN* | 4683 | 0.073 |
| *TGFBR2* | 7048 | 0.072 |
| *BCAR1* | 9564 | 0.072 |
| *PBOV1* | 59351 | 0.072 |
| *MET* | 4233 | 0.072 |
| *PRKCA* | 5578 | 0.07 |
| *IL6* | 3569 | 0.07 |
| *SDC1* | 6382 | 0.069 |
| *NFKB1* | 4790 | 0.069 |
| *PTK2* | 5747 | 0.069 |
| *MAPK3* | 5595 | 0.068 |
| *LACRT* | 90070 | 0.068 |
| *FEN1* | 2237 | 0.068 |

The top 100 lung cancer genes identified by using the KnowledgeNet approach.

| Gene symbol | Entrez ID | Confidence score |
| --- | --- | --- |
| *EGFR* | 1956 | 2.69 |
| *GSTM1* | 2944 | 0.857 |
| *SKP2* | 6502 | 0.722 |
| *TP53* | 7157 | 0.684 |
| *CXCR4* | 7852 | 0.673 |
| *GSTP1* | 2950 | 0.619 |
| *CYP1A1* | 1543 | 0.568 |
| *ERBB2* | 2064 | 0.533 |
| *RASSF1* | 11186 | 0.462 |
| *CADM1* | 23705 | 0.445 |
| *MPO* | 4353 | 0.404 |
| *PTGS2* | 5743 | 0.343 |
| *CDKN2A* | 1029 | 0.343 |
| *IGFBP3* | 3486 | 0.329 |
| *KRAS* | 3845 | 0.306 |
| *IL1B* | 3553 | 0.305 |
| *GSTT1* | 2952 | 0.29 |
| *BIRC3* | 330 | 0.287 |
| *BIRC2* | 329 | 0.286 |
| *MMP2* | 4313 | 0.244 |
| *XIAP* | 331 | 0.235 |
| *FHIT* | 2272 | 0.229 |
| *KRT8* | 3856 | 0.229 |
| *VEGFA* | 7422 | 0.22 |
| *BCL2* | 596 | 0.219 |
| *OGG1* | 4968 | 0.217 |
| *CYP2A13* | 1553 | 0.21 |
| *PLAUR* | 5329 | 0.205 |
| *PLAU* | 5328 | 0.205 |
| *LGALS3* | 3958 | 0.205 |
| *CDH1* | 999 | 0.2 |
| *FASN* | 2194 | 0.189 |
| *MGMT* | 4255 | 0.188 |
| *NQO1* | 1728 | 0.185 |
| *RALBP1* | 10928 | 0.183 |
| *ING1* | 3621 | 0.183 |
| *LGALS3BP* | 3959 | 0.182 |
| *SEMA3B* | 7869 | 0.17 |
| *IGF1* | 3479 | 0.169 |
| *FAS* | 355 | 0.167 |
| *IL8* | 3576 | 0.166 |
| *MYO18B* | 84700 | 0.161 |
| *CDKN1B* | 1027 | 0.155 |
| *CTNNB1* | 1499 | 0.154 |
| *GRP* | 2922 | 0.154 |
| *ASCL1* | 429 | 0.15 |
| *SLPI* | 6590 | 0.146 |
| *NKX2-1* | 7080 | 0.145 |
| *AREG* | 374 | 0.144 |
| *CDH13* | 1012 | 0.142 |
| *MET* | 4233 | 0.142 |
| *SOCS3* | 9021 | 0.142 |
| *ERCC2* | 2068 | 0.14 |
| *SFTPB* | 6439 | 0.14 |
| *CXCL12* | 6387 | 0.138 |
| *CTAG2* | 30848 | 0.137 |
| *MAPK1* | 5594 | 0.137 |
| *MMP9* | 4318 | 0.137 |
| *CASP8* | 841 | 0.136 |
| *PTEN* | 5728 | 0.136 |
| *SMARCA4* | 6597 | 0.135 |
| *RBL2* | 5934 | 0.133 |
| *TUBB2A* | 7280 | 0.131 |
| *PRKCE* | 5581 | 0.129 |
| *ITGA9* | 3680 | 0.128 |
| *RHOA* | 387 | 0.127 |
| *MAGEC2* | 51438 | 0.124 |
| *FEN1* | 2237 | 0.123 |
| *COX17* | 10063 | 0.116 |
| *ABCG2* | 9429 | 0.115 |
| *VEGFC* | 7424 | 0.113 |
| *FGF2* | 2247 | 0.108 |
| *RBM6* | 10180 | 0.108 |
| *PRKCA* | 5578 | 0.108 |
| *CDKN2B* | 1030 | 0.106 |
| *TYMS* | 7298 | 0.105 |
| *THPO* | 7066 | 0.104 |
| *DLC1* | 10395 | 0.103 |
| *ELAVL4* | 1996 | 0.102 |
| *JUP* | 3728 | 0.102 |
| *TOP1* | 7150 | 0.101 |
| *TSPYL2* | 64061 | 0.1 |
| *PLUNC* | 51297 | 0.099 |
| *CTSB* | 1508 | 0.099 |
| *CSF2* | 1437 | 0.098 |
| *TOP2A* | 7153 | 0.097 |
| *RARB* | 5915 | 0.096 |
| *NME1* | 4830 | 0.095 |
| *MYC* | 4609 | 0.094 |
| *SFTPD* | 6441 | 0.093 |
| *CAV1* | 857 | 0.091 |
| *XRCC1* | 7515 | 0.091 |
| *IL10* | 3586 | 0.089 |
| *MVP* | 9961 | 0.088 |
| *UBA7* | 7318 | 0.088 |
| *AKR1C1* | 1645 | 0.088 |
| *ADH5* | 128 | 0.086 |
| *KIT* | 3815 | 0.086 |
| *TXN* | 7295 | 0.086 |
| *ALDH3A1* | 218 | 0.085 |
