# Supplementary material for: Building a Statistical Model for Predicting Cancer Genes
Source: PLoS One. 2012 Nov 15;7(11):e49175. doi: 10.1371/journal.pone.0049175 (PMC3499550; doi:10.1371/journal.pone.0049175)
Supplement: Table S3 — Univariable analysis identified 22 of the original 33 original variables as significant predictors of PCa genes. (DOCX) [file pone.0049175.s003.docx]

| Supplementary Table S3. Univariate analysis of the original 33 predictors of PCa genes. | | | | |
| --- | --- | --- | --- | --- |
|  |  |  |  |  |
| Variable | Score | df | Sig. |  |
| Prostate specific expression (enrichment score) | 255.599 | 1 | <0.001 |  |
| Expression in normal prostate | 112.234 | 1 | <0.002 |  |
| Growth factors | 86.429 | 1 | <0.003 |  |
| Variance in tumor tissue | 80.737 | 1 | <0.004 |  |
| Phosphatases | 69.288 | 1 | <0.005 |  |
| Kinases | 68.901 | 1 | <0.006 |  |
| Variance in adjacent tissue | 55.876 | 1 | <0.007 |  |
| Anti apoptotic | 50.338 | 1 | <0.008 |  |
| Cell proliferation | 45.31 | 1 | <0.009 |  |
| Extracellular space | 42.298 | 1 | <0.010 |  |
| Signal transduction | 41.14 | 1 | <0.011 |  |
| Meta-analysis of the gene expression | 37.678 | 1 | <0.012 |  |
| Angiogenesis | 37.234 | 1 | <0.013 |  |
| Secreted | 30.551 | 1 | <0.014 |  |
| 3-level meta-analysis | 29.999 | 1 | <0.015 |  |
| Sumoylated | 19.95 | 1 | <0.016 |  |
| Difference in expression -LOG(P) | 15.606 | 1 | <0.017 |  |
| Mean expression in adjacent tissue | 12.667 | 1 | <0.018 |  |
| Mean expression in tumor tissue | 11.867 | 1 | 0.001 |  |
| Cell adhesion | 10.802 | 1 | 0.001 |  |
| Transcription factors | 10.394 | 1 | 0.001 |  |
| Plasma membrane | 9.427 | 1 | 0.002 |  |
| DNA repair | 3.648 | 1 | 0.056 |  |
| Ubiquitinated | 2.039 | 1 | 0.153 |  |
| Translation | 1.56 | 1 | 0.212 |  |
| Evolutionary conservation index | 0.864 | 1 | 0.352 |  |
| Acetylated | 0.751 | 1 | 0.386 |  |
| Chromatin remodeling | 0.326 | 1 | 0.568 |  |
| Transcription | 0.249 | 1 | 0.618 |  |
| Housekeeping gene | 0.061 | 1 | 0.805 |  |
| Phosphorylated | 0.053 | 1 | 0.817 |  |
| DNA replication | 0.011 | 1 | 0.917 |  |
| Methylated | 0.004 | 1 | 0.951 |  |
