# Supplementary material for: Building a Statistical Model for Predicting Cancer Genes
Source: PLoS One. 2012 Nov 15;7(11):e49175. doi: 10.1371/journal.pone.0049175 (PMC3499550; doi:10.1371/journal.pone.0049175)
Supplement: Table S4 — Ranking of the top 200 genes by model-generated probability of being PCa related. P, putative PCa gene; K, known PCa gene; NP, novel predicted PCa gene. (DOCX) [file pone.0049175.s004.docx]

|  |  |  |  |  |  |  |  |  |  |  |
| --- | --- | --- | --- | --- | --- | --- | --- | --- | --- | --- |
| Supplementary Table S4. Ranking of the top 400 genes by model-generated probability of being  PCa related. P, putative PCa gene; K, known PCa gene; NP, novel predicted PCa gene. | | | | | | | | | | |
|  |  |  |  |  |  |  |  |  |  |  |
|  |  |  |  |  |  |  |  |  |  |  |
| Rank | Gene Symbol | Entrez gene ID | Type of gene | Probability to be PCa |  |  |  |  |  |  |
| 1 | *TGM4* | 7047 | P | 0.992089015 |  |  |  |  |  |  |
| 2 | *RLN1* | 6013 | P | 0.982089015 |  |  |  |  |  |  |
| 3 | *SEMG1* | 6406 | P | 0.971647538 |  |  |  |  |  |  |
| 4 | *KLK3* | 354 | K | 0.904972121 |  |  |  |  |  |  |
| 5 | *KLK2* | 3817 | K | 0.888379948 |  |  |  |  |  |  |
| 6 | *TP53* | 7157 | K | 0.851598247 |  |  |  |  |  |  |
| 7 | *AR* | 367 | K | 0.849337452 |  |  |  |  |  |  |
| 8 | *PRAC* | 84366 | P | 0.827909443 |  |  |  |  |  |  |
| 9 | *UPK3A* | 7380 | NP | 0.822056318 |  |  |  |  |  |  |
| 10 | *OR51E2* | 81285 | K | 0.79784983 |  |  |  |  |  |  |
| 11 | *ACPP* | 55 | K | 0.788691371 |  |  |  |  |  |  |
| 12 | *HOXB13* | 10481 | P | 0.769586154 |  |  |  |  |  |  |
| 13 | *VEGFA* | 7422 | K | 0.760396348 |  |  |  |  |  |  |
| 14 | *NKX3-1* | 4824 | K | 0.708140636 |  |  |  |  |  |  |
| 15 | *BCAR1* | 9564 | P | 0.677467316 |  |  |  |  |  |  |
| 16 | *KITLG* | 4254 | NP | 0.667743972 |  |  |  |  |  |  |
| 17 | *NPY* | 4852 | NP | 0.6037839 |  |  |  |  |  |  |
| 18 | *CDH2* | 1000 | P | 0.561327407 |  |  |  |  |  |  |
| 19 | *GHR* | 2690 | NP | 0.551519488 |  |  |  |  |  |  |
| 20 | *SCGB1A* | 7356 | NP | 0.535101066 |  |  |  |  |  |  |
| 21 | *TRPM8* | 79054 | P | 0.502474718 |  |  |  |  |  |  |
| 22 | *SMAD3* | 4088 | P | 0.453467749 |  |  |  |  |  |  |
| 23 | *SLC45A* | 85414 | P | 0.451978169 |  |  |  |  |  |  |
| 24 | *NR3C1* | 2908 | NP | 0.433523502 |  |  |  |  |  |  |
| 25 | *TMPRSS* | 7113 | K | 0.425294271 |  |  |  |  |  |  |
| 26 | *IGF1* | 3479 | K | 0.422733459 |  |  |  |  |  |  |
| 27 | *TGFA* | 7039 | NP | 0.415828443 |  |  |  |  |  |  |
| 28 | *HIF1A* | 3091 | K | 0.407593528 |  |  |  |  |  |  |
| 29 | *PIK3R1* | 5295 | P | 0.391973921 |  |  |  |  |  |  |
| 30 | *STAT3* | 6774 | K | 0.382505074 |  |  |  |  |  |  |
| 31 | *JUP* | 3728 | NP | 0.375701317 |  |  |  |  |  |  |
| 32 | *NPM1* | 4869 | NP | 0.36077898 |  |  |  |  |  |  |
| 33 | *CD177* | 57126 | NP | 0.357430246 |  |  |  |  |  |  |
| 34 | *FAM55D* | 54827 | NP | 0.355934734 |  |  |  |  |  |  |
| 35 | *TGFB2* | 7042 | P | 0.355511042 |  |  |  |  |  |  |
| 36 | *GDF15* | 9518 | K | 0.353627664 |  |  |  |  |  |  |
| 37 | *MAP3K5* | 4217 | P | 0.351790125 |  |  |  |  |  |  |
| 38 | *TCF7L2* | 6934 | P | 0.34957372 |  |  |  |  |  |  |
| 39 | *XCL2* | 6846 | NP | 0.349010274 |  |  |  |  |  |  |
| 40 | *GNB2L1* | 10399 | NP | 0.347270528 |  |  |  |  |  |  |
| 41 | *MAPK1* | 5594 | P | 0.337405575 |  |  |  |  |  |  |
| 42 | *CCL2* | 6347 | NP | 0.329939252 |  |  |  |  |  |  |
| 43 | *FLNA* | 2316 | P | 0.32944222 |  |  |  |  |  |  |
| 44 | *ANGPT1* | 284 | NP | 0.329224421 |  |  |  |  |  |  |
| 45 | *EGFR* | 1956 | K | 0.324122055 |  |  |  |  |  |  |
| 46 | *ERBB2* | 2064 | K | 0.32354659 |  |  |  |  |  |  |
| 47 | *CXCL1* | 2919 | NP | 0.318773889 |  |  |  |  |  |  |
| 48 | *SOD1* | 6647 | NP | 0.317269314 |  |  |  |  |  |  |
| 49 | *DAB2IP* | 153090 | K | 0.316115782 |  |  |  |  |  |  |
| 50 | *AKT1* | 207 | P | 0.308032996 |  |  |  |  |  |  |
| 51 | *NDP* | 4693 | NP | 0.299291178 |  |  |  |  |  |  |
| 52 | *CTNNB1* | 1499 | P | 0.295629822 |  |  |  |  |  |  |
| 53 | *IGF1R* | 3480 | P | 0.294838891 |  |  |  |  |  |  |
| 54 | *AMACR* | 23600 | K | 0.294429284 |  |  |  |  |  |  |
| 55 | *FGF6* | 2251 | NP | 0.287128381 |  |  |  |  |  |  |
| 56 | *MAP2K7* | 5609 | NP | 0.280768774 |  |  |  |  |  |  |
| 57 | *CALM1* | 801 | NP | 0.27622261 |  |  |  |  |  |  |
| 58 | *RARA* | 5914 | P | 0.271122459 |  |  |  |  |  |  |
| 59 | *AMELX* | 265 | NP | 0.265231822 |  |  |  |  |  |  |
| 60 | *TP63* | 8626 | P | 0.261539016 |  |  |  |  |  |  |
| 61 | *AKAP1* | 8165 | NP | 0.256916213 |  |  |  |  |  |  |
| 62 | *PCDHB8* | 56128 | NP | 0.249966587 |  |  |  |  |  |  |
| 63 | *FAS* | 355 | P | 0.246684688 |  |  |  |  |  |  |
| 64 | *IL1B* | 3553 | NP | 0.245771114 |  |  |  |  |  |  |
| 65 | *OR51E1* | 143503 | NP | 0.245415676 |  |  |  |  |  |  |
| 66 | *PCDHB1* | 56123 | NP | 0.23941096 |  |  |  |  |  |  |
| 67 | *ATF7* | 11016 | NP | 0.225237845 |  |  |  |  |  |  |
| 68 | *TRAF6* | 7189 | NP | 0.224469835 |  |  |  |  |  |  |
| 69 | *EPAS1* | 2034 | P | 0.22337745 |  |  |  |  |  |  |
| 70 | *FOLH1* | 2346 | K | 0.218640713 |  |  |  |  |  |  |
| 71 | *TBX3* | 6926 | NP | 0.216531548 |  |  |  |  |  |  |
| 72 | *DLG1* | 1739 | NP | 0.216432094 |  |  |  |  |  |  |
| 73 | *EGF* | 1950 | P | 0.216082357 |  |  |  |  |  |  |
| 74 | *IL2* | 3558 | NP | 0.208855973 |  |  |  |  |  |  |
| 75 | *GSTP1* | 2950 | K | 0.201123661 |  |  |  |  |  |  |
| 76 | *HSP90B* | 7184 | NP | 0.197168701 |  |  |  |  |  |  |
| 77 | *NRG1* | 3084 | NP | 0.189905843 |  |  |  |  |  |  |
| 78 | *MET* | 4233 | K | 0.184953803 |  |  |  |  |  |  |
| 79 | *ANXA1* | 301 | NP | 0.181895827 |  |  |  |  |  |  |
| 80 | *BRAF* | 673 | P | 0.181245421 |  |  |  |  |  |  |
| 81 | *CXCL12* | 6387 | K | 0.175115851 |  |  |  |  |  |  |
| 82 | *THBS1* | 7057 | P | 0.173639803 |  |  |  |  |  |  |
| 83 | *BCL2* | 596 | K | 0.170487634 |  |  |  |  |  |  |
| 84 | *SRD5A2* | 6716 | K | 0.168630996 |  |  |  |  |  |  |
| 85 | *FGF9* | 2254 | NP | 0.166516708 |  |  |  |  |  |  |
| 86 | *SMAD1* | 4086 | NP | 0.164830847 |  |  |  |  |  |  |
| 87 | *AKAP11* | 11215 | NP | 0.164637842 |  |  |  |  |  |  |
| 88 | *LTF* | 4057 | NP | 0.160940103 |  |  |  |  |  |  |
| 89 | *TFF1* | 7031 | NP | 0.157846395 |  |  |  |  |  |  |
| 90 | *ARTN* | 9048 | NP | 0.15652501 |  |  |  |  |  |  |
| 91 | *PTEN* | 5728 | K | 0.154678673 |  |  |  |  |  |  |
| 92 | *TMEFF2* | 23671 | P | 0.153730409 |  |  |  |  |  |  |
| 93 | *HDGF* | 3068 | NP | 0.151678216 |  |  |  |  |  |  |
| 94 | *RELA* | 5970 | NP | 0.150328696 |  |  |  |  |  |  |
| 95 | *GDNF* | 2668 | NP | 0.149980694 |  |  |  |  |  |  |
| 96 | *VEGFC* | 7424 | P | 0.144845313 |  |  |  |  |  |  |
| 97 | *SPRED1* | 161742 | NP | 0.140801228 |  |  |  |  |  |  |
| 98 | *SYK* | 6850 | NP | 0.140470229 |  |  |  |  |  |  |
| 99 | *MEF2C* | 4208 | NP | 0.135963905 |  |  |  |  |  |  |
| 100 | *VHL* | 7428 | NP | 0.134869944 |  |  |  |  |  |  |
| 101 | *PTK2B* | 2185 | P | 0.131895248 |  |  |  |  |  |  |
| 102 | *TNFRSF* | 8793 | P | 0.129674746 |  |  |  |  |  |  |
| 103 | *NELL1* | 4745 | NP | 0.12910794 |  |  |  |  |  |  |
| 104 | *LGALS3* | 3959 | NP | 0.128137956 |  |  |  |  |  |  |
| 105 | *INSR* | 3643 | NP | 0.127780476 |  |  |  |  |  |  |
| 106 | *ITGAV* | 3685 | K | 0.125629461 |  |  |  |  |  |  |
| 107 | *HSPB1* | 3315 | NP | 0.124376983 |  |  |  |  |  |  |
| 108 | *CTGF* | 1490 | NP | 0.124304473 |  |  |  |  |  |  |
| 109 | *CPLX3* | 594855 | NP | 0.123681433 |  |  |  |  |  |  |
| 110 | *SKI* | 6497 | NP | 0.123036997 |  |  |  |  |  |  |
| 111 | *SOX9* | 6662 | P | 0.121519872 |  |  |  |  |  |  |
| 112 | *IGF2* | 3481 | NP | 0.121484229 |  |  |  |  |  |  |
| 113 | *WNT5B* | 81029 | NP | 0.118899824 |  |  |  |  |  |  |
| 114 | *FOXO1* | 2308 | P | 0.117856899 |  |  |  |  |  |  |
| 115 | *MAPK6* | 5597 | NP | 0.117207315 |  |  |  |  |  |  |
| 116 | *AZGP1* | 563 | P | 0.116496105 |  |  |  |  |  |  |
| 117 | *PROK2* | 60675 | NP | 0.115668006 |  |  |  |  |  |  |
| 118 | *THBS4* | 7060 | NP | 0.114869886 |  |  |  |  |  |  |
| 119 | *NFKB1* | 4790 | K | 0.114820508 |  |  |  |  |  |  |
| 120 | *GPI* | 2821 | NP | 0.113689551 |  |  |  |  |  |  |
| 121 | *ACVRL1* | 94 | NP | 0.113347927 |  |  |  |  |  |  |
| 122 | *GFER* | 2671 | NP | 0.111080414 |  |  |  |  |  |  |
| 123 | *PPP1R1* | 4659 | NP | 0.110565376 |  |  |  |  |  |  |
| 124 | *GSK3B* | 2932 | K | 0.10992585 |  |  |  |  |  |  |
| 125 | *BMP5* | 653 | K | 0.109775905 |  |  |  |  |  |  |
| 126 | *JAM3* | 83700 | NP | 0.109764417 |  |  |  |  |  |  |
| 127 | *ZNF532* | 55205 | NP | 0.10795508 |  |  |  |  |  |  |
| 128 | *PDGFD* | 80310 | NP | 0.106830619 |  |  |  |  |  |  |
| 129 | *TYRO3* | 7301 | NP | 0.106603101 |  |  |  |  |  |  |
| 130 | *MIA* | 8190 | NP | 0.10457002 |  |  |  |  |  |  |
| 131 | *PGF* | 5228 | NP | 0.104150745 |  |  |  |  |  |  |
| 132 | *TEPP* | 374739 | NP | 0.103026367 |  |  |  |  |  |  |
| 133 | *PPP3CB* | 5532 | NP | 0.102881485 |  |  |  |  |  |  |
| 134 | *ID1* | 3397 | K | 0.10268499 |  |  |  |  |  |  |
| 135 | *COMP* | 1311 | NP | 0.102236492 |  |  |  |  |  |  |
| 136 | *AMBN* | 258 | NP | 0.101464757 |  |  |  |  |  |  |
| 137 | *PSCA* | 8000 | K | 0.101098028 |  |  |  |  |  |  |
| 138 | *SATB1* | 6304 | NP | 0.100549552 |  |  |  |  |  |  |
| 139 | *COL4A6* | 1288 | NP | 0.100002717 |  |  |  |  |  |  |
| 140 | *ANG* | 283 | NP | 0.099632336 |  |  |  |  |  |  |
| 141 | *ILK* | 3611 | P | 0.098457171 |  |  |  |  |  |  |
| 142 | *FGF18* | 8817 | NP | 0.09820261 |  |  |  |  |  |  |
| 143 | *IKZF1* | 10320 | NP | 0.097741 |  |  |  |  |  |  |
| 144 | *SIRT1* | 23411 | NP | 0.097438276 |  |  |  |  |  |  |
| 145 | *IRS1* | 3667 | NP | 0.097311175 |  |  |  |  |  |  |
| 146 | *GRN* | 2896 | P | 0.096802744 |  |  |  |  |  |  |
| 147 | *ZNF35* | 7584 | NP | 0.096709097 |  |  |  |  |  |  |
| 148 | *BMP2* | 650 | K | 0.095181382 |  |  |  |  |  |  |
| 149 | *TRPS1* | 7227 | K | 0.095105088 |  |  |  |  |  |  |
| 150 | *FOS* | 2353 | NP | 0.094401236 |  |  |  |  |  |  |
| 151 | *MEF2A* | 4205 | NP | 0.093684841 |  |  |  |  |  |  |
| 152 | *SCUBE2* | 57758 | NP | 0.093236115 |  |  |  |  |  |  |
| 153 | *FN1* | 2335 | NP | 0.093142126 |  |  |  |  |  |  |
| 154 | *FOLH1B* | 219595 | NP | 0.092927583 |  |  |  |  |  |  |
| 155 | *TGFBI* | 7045 | K | 0.09200105 |  |  |  |  |  |  |
| 156 | *ACTG2* | 72 | P | 0.091120885 |  |  |  |  |  |  |
| 157 | *CFB* | 629 | NP | 0.08997944 |  |  |  |  |  |  |
| 158 | *PTN* | 5764 | NP | 0.089975352 |  |  |  |  |  |  |
| 159 | *AURKA* | 6790 | NP | 0.088685611 |  |  |  |  |  |  |
| 160 | *ADAM9* | 8754 | NP | 0.088095743 |  |  |  |  |  |  |
| 161 | *ANAPC5* | 51433 | NP | 0.087782233 |  |  |  |  |  |  |
| 162 | *CSTA* | 1475 | NP | 0.087662175 |  |  |  |  |  |  |
| 163 | *PLA2G2* | 5320 | P | 0.087272768 |  |  |  |  |  |  |
| 164 | *BAD* | 572 | P | 0.087167986 |  |  |  |  |  |  |
| 165 | *DKK1* | 22943 | NP | 0.086766219 |  |  |  |  |  |  |
| 166 | *BCL2L1* | 598 | K | 0.086119794 |  |  |  |  |  |  |
| 167 | *SFN* | 2810 | P | 0.085686548 |  |  |  |  |  |  |
| 168 | *IFI16* | 3428 | P | 0.085503243 |  |  |  |  |  |  |
| 169 | *FGFR2* | 2263 | P | 0.085322631 |  |  |  |  |  |  |
| 170 | *IL6* | 3569 | K | 0.084704458 |  |  |  |  |  |  |
| 171 | *IL10* | 3586 | P | 0.08460792 |  |  |  |  |  |  |
| 172 | *DPP4* | 1803 | P | 0.084570524 |  |  |  |  |  |  |
| 173 | *SPON2* | 10417 | NP | 0.084004307 |  |  |  |  |  |  |
| 174 | *NCAPD3* | 23310 | NP | 0.083968076 |  |  |  |  |  |  |
| 175 | *TRIP6* | 7205 | NP | 0.083930704 |  |  |  |  |  |  |
| 176 | *SPHK1* | 8877 | NP | 0.08361003 |  |  |  |  |  |  |
| 177 | *SREBF1* | 6720 | P | 0.083398046 |  |  |  |  |  |  |
| 178 | *JAG1* | 182 | NP | 0.083262413 |  |  |  |  |  |  |
| 179 | *CCNL2* | 81669 | NP | 0.08303693 |  |  |  |  |  |  |
| 180 | *ALOX15* | 247 | K | 0.082870745 |  |  |  |  |  |  |
| 181 | *MYH11* | 4629 | P | 0.082252992 |  |  |  |  |  |  |
| 182 | *DUSP2* | 1844 | NP | 0.081557226 |  |  |  |  |  |  |
| 183 | *VEGFB* | 7423 | NP | 0.081114555 |  |  |  |  |  |  |
| 184 | *MAP2K1* | 5604 | P | 0.081077372 |  |  |  |  |  |  |
| 185 | *GAS6* | 2621 | NP | 0.080966095 |  |  |  |  |  |  |
| 186 | *CLU* | 1191 | P | 0.08076999 |  |  |  |  |  |  |
| 187 | *FGF2* | 2247 | P | 0.080510077 |  |  |  |  |  |  |
| 188 | *IGFBP4* | 3487 | NP | 0.079622113 |  |  |  |  |  |  |
| 189 | *TRAF4* | 9618 | P | 0.079235534 |  |  |  |  |  |  |
| 190 | *CREM* | 1390 | NP | 0.078598485 |  |  |  |  |  |  |
| 191 | *PDLIM5* | 10611 | NP | 0.078171188 |  |  |  |  |  |  |
| 192 | *JUN* | 3725 | P | 0.07805322 |  |  |  |  |  |  |
| 193 | *GREM1* | 26585 | NP | 0.077875025 |  |  |  |  |  |  |
| 194 | *PDGFC* | 56034 | NP | 0.077647052 |  |  |  |  |  |  |
| 195 | *LAMA5* | 3911 | NP | 0.077551339 |  |  |  |  |  |  |
| 196 | *MANF* | 7873 | P | 0.076805954 |  |  |  |  |  |  |
| 197 | *CD8A* | 925 | NP | 0.076802552 |  |  |  |  |  |  |
| 198 | *YAP1* | 10413 | NP | 0.07564943 |  |  |  |  |  |  |
| 199 | *MAP3K1* | 4214 | P | 0.075347394 |  |  |  |  |  |  |
| 200 | *CD4* | 920 | NP | 0.075333971 |  |  |  |  |  |  |
