# Supplementary material for: Building a Statistical Model for Predicting Cancer Genes
Source: PLoS One. 2012 Nov 15;7(11):e49175. doi: 10.1371/journal.pone.0049175 (PMC3499550; doi:10.1371/journal.pone.0049175)
Supplement: Information S1 — Description of the Variables Used to Build the Prediction Model. Variables Are Listed in the Order in Which They Are Presented in Table 1. (DOCX) [file pone.0049175.s006.docx]

**Supporting information S1.**

**Description of the Variables Used to Build the Prediction Model. Variables Are Listed in the Order in Which They Are Presented in Table 1.**

*Three-level meta-analysis* (gene expression, continuous variable): Data from our meta-analysis of the genes differently expressed in prostate cancer (Gorlov et al. 2010). We have used absolute values of *Z* scores from the meta-analysis. The higher the *Z*, the stronger the evidence that the expression of the gene changes in prostate tumorigenesis.

*Acetylated* (postranslational modifications, binary variable): Data from GO: The variable equals 1 when at least one protein encoded by the gene is reported as acetylated by GO and 0 if there is no report that at least one protein encoded by the gene is acetylated.

*Angiogenesis* (biologic function, binary variable): Data from GO: The variable equals 1 when the gene is reported as associated with angiogenesis and 0 if there is no report that the gene plays a role in angiogenesis.

*Antiapoptotic* (biologic function, binary variable): Data from GO: The variable equals 1 when the gene plays a role in antiapoptotic response and 0 if there is no report that the gene is antiapoptotic.

*Cell adhesion* (biologic function, binary variable): Data from GO: The variable equals 1 when the gene is involved in cell adhesion and 0 if there is no report that the gene is associated with cell adhesion.

*Cell proliferation* (biologic function, binary variable): Data from GO: The variable equals 1 when the gene is involved in cell proliferation and 0 if there is no report that the gene is associated with cell proliferation.

*Chromatin remodeling* (biologic function, binary variable): Data from GO: The variable equals 1 when the gene is involved in chromatin remodeling and 0 if there is no report that the gene is associated with chromatin remodeling.

*Difference in expression –LOG(P)* (gene expression, continuous variable): Average Student’s *t* test value from two published studies on gene expression in primary prostate tumor and adjacent normal tissues (Chandran et al. 2007; Taylor et al. 2010). In our preliminary analysis, we found that the results of these studies are consistent, which is why we combined them.

*DNA repair* (biologic function, binary variable): Data from GO: The variable equals 1 when the gene is involved in DNA repair and 0 if there is no report that the gene is associated with DNA repair.

*DNA replication* (biologic function, binary variable): Data from GO: The variable equals 1 when the gene is involved in DNA replication and 0 if there is no report that the gene is associated with DNA replication.

*Evolutionary conservation index* (level of evolutionary conservation of the gene, continuous variable): Number of orthologs (copies of the gene found in other species) was used as a measure of evolutionary conservation, similarly to the way it was done in other studies (Huang et al. 2004; Choi et al. 2007). The HomoloGene database was used to retrieve the data on the number of orthologs.

## *Expression level in normal prostate* (gene expression, continuous variable): The Expressed Sequence Tag (EST)–based assessment of the gene in normal human prostate tissue. Data from the Tissue-specific Gene Expression and Regulation (TiGER) database: <http://bioinfo.wilmer.jhu.edu/tiger/>.

## *Extracellular space* (cellular localization, binary variable): Data from GO: The variable equals 1 when the gene’s product is located in the extracellular space and 0 if there is no report that the gene’s product is located in the extracellular space.

## *Growth factors* (type of protein, binary variable): Data from GO: The variable equals 1 when the gene is a growth factor and 0 if there is no report that the gene is a growth factor.

*Housekeeping gene* (gene category, binary variable): We used the list of housekeeping genes from the paper by Eisenberg and Levanon (2003). The variable equals 1 if the gene is a housekeeping gene and 0 if the gene is not a housekeeping gene.

*Kinases* (type of protein, binary variable): Data from GO: The variable equals 1 when the gene is a kinase and 0 if there is no report that the gene is a kinase.

*Mean expression in adjacent tissue* (gene expression, continuous variable): Weighted by sample size, mean gene expression values in adjacent normal tissue: Combined data from Chandran et al. (2007) and Taylor et al. (2010).

*Mean expression in tumor tissue* (gene expression, continuous variable): Mean gene expression values in primary prostate tumors: Data from Chandran et al. (2007) and Taylor et al. (2010).

*Meta-analysis of gene expression* (gene expression, continuous variable): Data from our meta-analysis of gene expression in the primary prostate tumor vs. adjacent normal tissue (Gorlov et al. 2009). Meta-analysis–derived *Z* scores were used as a quantitative measure of the differences in gene expression.

*Methylated* (posttranslational modifications, binary variable): Data from GO: The variable equals 1 when at least one protein encoded by the gene is reported as methylated and 0 if there is no report that at least one protein encoded by the gene is methylated.

*Phosphatases* (type of protein, binary variable): Data from GO: The variable equals 1 when the gene is a phosphatase and 0 if there is no report that the gene is a phosphatase.

*Phosphorylated* (posttranslational modifications, binary variable): Data from GO: The variable equals 1 when at least one protein encoded by the gene is reported as phosphorylated.

*Plasma membrane* (cellular localization, binary variable): Data from GO: The variable equals 1 when the gene’s product is located in the plasma membrane and is 0 if there is no report that the gene’s product is located in the plasma membrane.

## *Prostate-specific expression (enrichment score)* (tissue specificity of the gene expression, continuous variable): Data from the Tissue-specific Gene Expression and Regulation (TiGER) database: <http://bioinfo.wilmer.jhu.edu/tiger/>. We used the enrichment score for the prostate: the higher the enrichment score, the more prostate specific the expression of the gene. Details on calculation of the enrichment score can be found in Liu et al. (2008).

*Secreted* (cellular localization, binary variable): Data from GO: The variable equals 1 when the gene’s product is reported as secreted and 0 if there is no report that the gene’s product is secreted.

*Signal transduction* (biologic function, binary variable): Data from GO: The variable equals 1 when the gene is involved in signal transduction and 0 if there is no report that the gene is involved in signal transduction.

*Sumoylated* (posttranslational modifications, binary variable): Data from GO: The variable equals 1 when the protein encoded by the gene is reported as sumoylated. Sumoylation is the process of attachment to the protein of Small Ubiquitin-like Modifier (SUMO) molecules, which leads to ubiquitination.

*Transcription* (biologic function, binary variable): Data from GO: The variable equals 1 when the gene plays a role in transcription and 0 if there is no report that the gene plays a role in transcription.

*Transcription factors* (type of protein, binary variable): Data from GO: The variable equals 1 when the gene is reported as a transcription factor and 0 if there is no report that the gene is a transcription factor.

*Translation* (biologic function, binary variable): Data from GO: The variable equals 1 when the gene plays a role in translation and 0 if there is no report that the gene plays a role in translation.

*Ubiquitinated* (posttranslational modifications, binary variable): Data from GO: The variable equals 1 when the protein encoded by the gene is reported as ubiquitinated.

*Variance in adjacent tissue* (gene expression, continuous variable): Mean interindividual variance of the gene expression values in adjacent normal tissue: Combined data from Chandran et al. (2007) and Taylor et al. (2010).

*Variance in tumor tissue* (gene expression, continuous variable): Mean interindividual variance of the gene expression values in tumor tissue: Combined data from Chandran et al. (2007) and Taylor et al. (2010).

**References**

Chandran UR, Ma C, Dhir R, Bisceglia M, Lyons-Weiler M et al. (2007) Gene expression profiles of prostate cancer reveal involvement of multiple molecular pathways in the metastatic process. BMC Cancer 7: 64.

Choi JK, Kim SC, Seo J, Kim S, Bhak J (2007) Impact of transcriptional properties on essentiality and evolutionary rate. Genetics 175(1): 199-206.

Eisenberg E, Levanon EY (2003) Human housekeeping genes are compact. Trends Genet 19(7): 362-365.

Gorlov IP, Byun J, Gorlova OY, Aparicio AM, Efstathiou E et al. (2009) Candidate pathways and genes for prostate cancer: a meta-analysis of gene expression data. BMC Med Genomics 2(1): 48.

Gorlov IP, Sircar K, Zhao H, Maity SN, Navone NM et al. (2010) Prioritizing genes associated with prostate cancer development. BMC Cancer 10: 599.

Huang H, Winter EE, Wang H, Weinstock KG, Xing H et al. (2004) Evolutionary conservation and selection of human disease gene orthologs in the rat and mouse genomes. Genome Biol 5(7): R47.

Liu X, Yu X, Zack DJ, Zhu H, Qian J (2008) TiGER: a database for tissue-specific gene expression and regulation. BMC Bioinformatics 9: 271.

Taylor BS, Schultz N, Hieronymus H, Gopalan A, Xiao Y et al. (2010) Integrative genomic profiling of human prostate cancer. Cancer Cell 18(1): 11-22.
